# Long-term maize-soybean crop rotation: impacts on soybean yield, soil microbiota and nitrogen dynamics

**DOI:** 10.3389/fpls.2025.1658885

**Published:** 2025-10-27

**Authors:** Liqiang Zhang, Jiamin Yin, Wenxiu Ji, Jingcheng Zhao, Zhuo Xin, Demin Rao, Fangang Meng, Jinhu Cui, Wei Zhang, Hongyan Zhao

**Affiliations:** ^1^ College of Agronomy, Yanbian University, Yanji, China; ^2^ College of Plant Science, Jilin University, Changchun, China; ^3^ Soybean Research Institute, Jilin Academy of Agricultural Sciences/National Engineering Research Center of Soybean, Changchun, China

**Keywords:** soybean maize rotation, nitrogen cycling, nitrogen form, soil microenvironment, yield

## Abstract

**Aims:**

Soil nitrogen is recognized as a vital nutrient influencing soybean growth and yield. Hence, a comprehensive understanding of the intricate connections between shifts in nitrogen patterns and the behaviors of soil microbial communities and crucial enzymes in the nitrogen cycle is highly desirable.

**Methods:**

This study involved a rotation positioning experiment spanning 11 years (2012-2022). Measurement of soil microenvironment changes during the mature period for three consecutive years (2020-2022). Based on these groups, the study delved into the alterations in nitrogen patterns within the soybean rotation, examining both soil enzyme activity and microbial community dynamics.

**Results:**

Long-term crop rotation and nitrogen application led to an increase ranging from 2.16% to 108.34% in the nine components of soil nitrogen. *Gemmatimonas*, *Rhodanobacter* and *Mrakia* could effectively increase soil nitrogen content and had a reciprocal promotion with soil urease and protease activities, whereas *Blastococcus* and *Fusarium* increased soil nitrogen loss. Changes in inorganic nitrogen and total organic nitrogen resulting from crop rotation enhanced the abundance of soil microbial communities, reducing their diversity.

**Conclusions:**

Overall, findings demonstrate that long-term crop rotation and nitrogen management significantly influence soil nitrogen dynamics, microbial community structure, and enzyme activities. Thus, enhancing the functional capacities of soil microbial communities to support sustainable soybean production.

## 1 Introduction

Leguminous crops inherently possess nitrogen-fixing capabilities, which provide nitrogen nutritional supplies for subsequent cereal crops. The root systems of leguminous crops play a significant role in shaping this soil microenvironment during their growth (Aparicio and Costa, 2007). The biological nitrogen fixation capacity of China’s soybean-growing areas can reach up to a maximum of 150 kg ha^-1^ (Sui and Yuan, 2023). In China’s intensive agricultural landscapes, the widespread deployment of high-nitrogen fertilizers constitutes 15% of the total arable land area (Han et al., 2025). This excessive application has led to poor nitrogen utilization efficiency, soil compaction, and a decrease in soil organic matter content. Simultaneously, Nitrogen that is not absorbed by plants enters the soil, atmosphere, and water bodies, polluting the ecological environment (Raza et al., 2019; Mori et al., 2023). Hence, establishing a green and sustainable crop rotation model is of paramount significance, especially in intensively cultivated areas.

Vance (2001) highlighted that 80–100 kg ha^-1^ of nitrogen in subsequent-season crops emanates from preceding-season leguminous cover crops (Vance, 2001). A three-year field experiment demonstrated that crop rotation with leguminous crops augmented maize yield and nitrogen utilization efficiency in the subsequent season by 35.5% and 33.0%, respectively (Gan et al., 2015). Similarly, maize yields in France exhibited an average annual decline of 0.035 t ha^-1^ over a 15-year span, when leguminous crop cultivation was reduced (Brisson et al., 2010). Hence, a comprehensive exploration of the impact of leguminous crops within crop rotation systems on nitrogen supply mechanisms for succeeding crops holds substantial practical implications. This endeavor is pivotal for reducing nitrogen inputs, enhancing nitrogen utilization efficiency, and producing crops with excellent quality and yield.

Soil nitrogen levels, forms, and transformations have a direct influence on the nutritional capacity of crops (Dessureault-Rompré, 2022). Inorganic nitrogen is primarily derived from residual soil sources as well as the mineralization of organic nitrogen from soil or applied organic materials (Heinzle et al., 2021). Soil acid hydrolyzable nitrogen, exchangeable ammonium, nitrate nitrogen, and alkaline hydrolyzable nitrogen constitute the main forms of nitrogen, comprising 2.61% to 14.85% of total nitrogen. These forms serve as the principal sources of nitrogen nutrition for soil plants, with direct absorption and assimilation (Anugroho and Kitou, 2020). Organic nitrogen accounts for more than 90% of total nitrogen in soil, representing the predominant nitrogen form. Its content and distribution are closely linked to soil organic matter (Soman et al., 2017). Labile organic nitrogen, a highly prevalent category of nitrogen compounds, typically constitutes 20% to 60% of total soil nitrogen (Chen et al., 2018). This category mainly resides within soil organic matter, including proteins and peptides (Crespo et al., 2021).

Soil hydrolysates also include light organic nitrogen, accounting for about 5% to 10% of total soil nitrogen (Leinweber et al., 2013). It generally exists in the form of polysaccharide structures and can interact with small molecules like antimicrobial substances in conjunction with sticky peptides and proteins. In soil, it potentially serves a dual role, acting as a source of energy for plant growth and assisting the development of a sound soil structure (Braos et al., 2016; Feng et al., 2018). Crop rotation enhances nutrient cycling, boosts soil organic carbon, and augments soil fertility. A rice-bean rotation system effectively mitigates continuous cropping obstacles, resulting in increased yields and better crop quality. Concurrently, a notable surge in both the abundance and diversity of subterranean microorganisms is observed, imparting a transformative enhancement to the intricate tapestry of soil microstructures (Hu et al., 2025).

The enzymatic activities of soil exhibit significant correlations with indicators such as soil nitrogen content and microbial communities (Sun et al., 2022). Within the realm of soil, enzymes such as urease, protease, and nitrate reductase exhibit enhanced activity (Park et al., 2016). A plethora of investigations have demonstrated crop rotation’s ability to foster microbial diversity in soil, while concomitantly bolstering enzymatic activity in the arable strata (Liu et al., 2024). According to Wang et al. (2025), conventional cultivation does not generate discernable discrepancies in soil enzymatic activity across varying soil depths, whereas rotated cultivation unveils a distinct stratification of enzyme activity in congruence with soil depth (Mori et al., 2023). Under divergent crop rotation regimes, the activity of distinct enzymes is variably influenced. Within the maize-soybean rotation paradigm, enzymes like soil urease, protease, and nitrate reductase demonstrate noteworthy distinctions in activity when compared to monoculture soybean and continuous maize scenarios (Lu et al., 2024).

The investigation of the complex interaction between crop rotation and the intricate tapestry of soil’s nitrogen-fixing bacterial communities has been predominantly guided by rudimentary methodologies, such as age-old breeding practices. Although traditional, these approaches fall short of capturing nuanced abundance, precise evaluation of nitrogen fixation, and the subtle shifts within bacterial populations in the intricate milieu of complex soil ecosystems (Wang et al., 2025). The higher the abundance of microorganisms such as bacteria, fungi, and actinomycetes in the soil, the greater the stability of their communities, thereby enhancing soil fertility and promoting plant growth (Sun et al., 2020). Continuous cropping has been shown to limit the amount of rhizobia and inhibit root nodule formation, thereby fostering the development of certain diseases (Li et al., 2018).

Prolonged crop rotation increases the population of Bacillus, *Streptomyces*, and *Acidobacteria* in the soil, whereas Bacillus, *Rhodococcus*, and yeast encourage crop growth and yield, ultimately improving the structure and functionality of the soil’s microbial community (Saijai et al., 2016). Similarly, Perez-Brandan et al. (2014) found that long-term continuous cropping of soybeans increased soil fungal and plant diseases, with fungal populations significantly lower in soybeans under crop rotation with rice (Perez-Brandan et al., 2014). Several studies have reported continuous cropping significantly reduces the quality of soybeans, the quantity and vitality of coexisting rhizobia, as well as nitrogen fixation. A 5-year soybean planting cycle induces substantial diversity shifts within nitrogen-fixing bacterial populations in the soil as compared to maize-soybean rotation. Different planting strategies reduce the types and quantities of nitrifying bacteria to varying degrees, accompanied by distinct alterations in the bacterial community structure of the soil (Narayana et al., 2022).

Building upon the aforementioned literature, we hypothesized that crop rotation improves the soil nitrogen content and crop yield through microorganism-mediated enhancement of the nitrogen cycle. The objectives of this study are to (1) explore the influence of long-term maize-soybean rotations coupled with nitrogen application on soil nitrogen forms, enzymatic activities, and microbial community structure; (2) elucidate the effects of soil enzymes and microbial communities on changes in various forms of nitrogen.

## 2 Materials and methods

### 2.1 Site profile and experimental design

**Figure 1:**
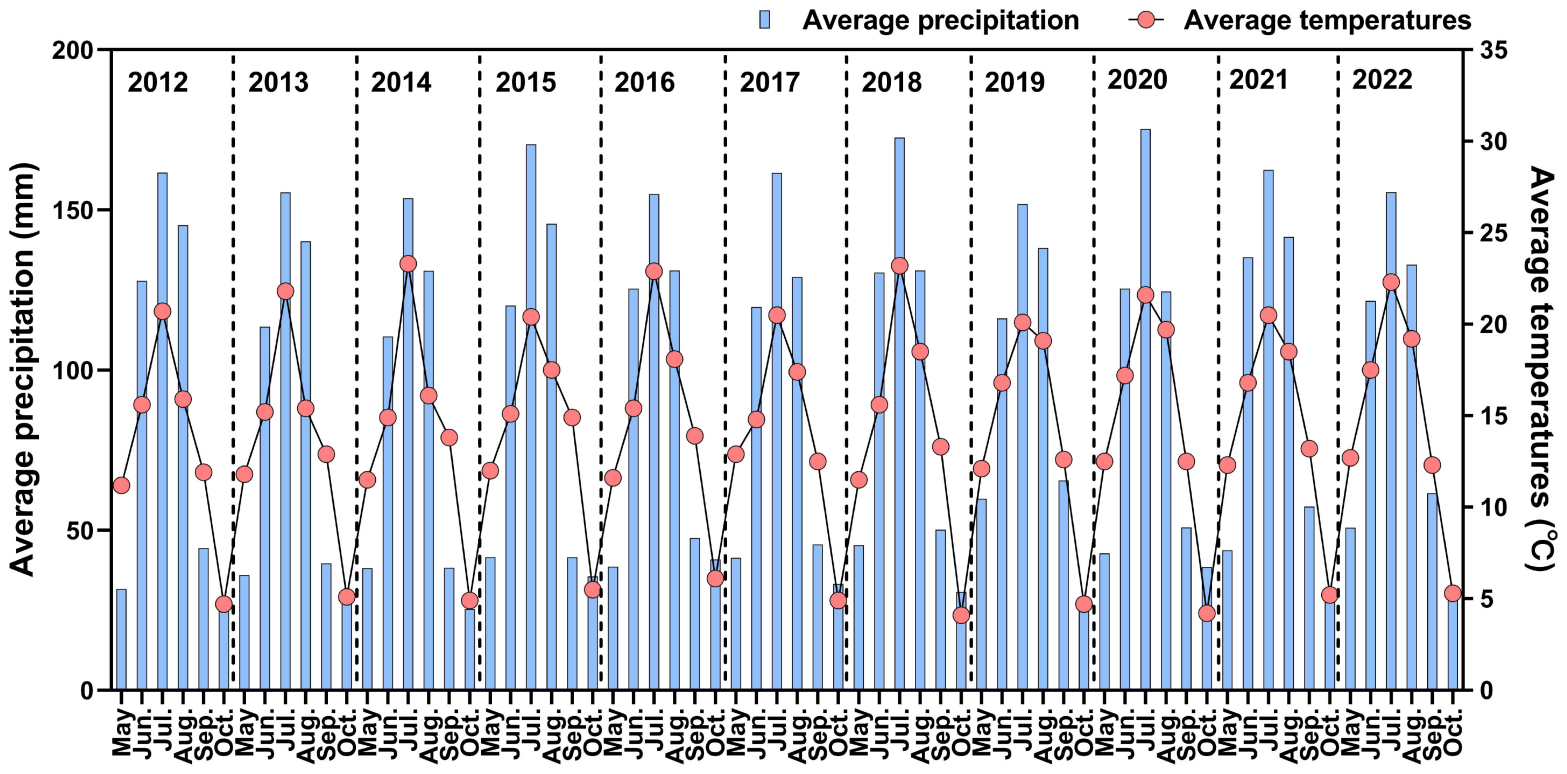
Monthly average rainfall and average temperature recorded during the maize and soybean experimental period (2012–2022).

A long-term field experiment was established in May 2012 with two systems: continuous soybean cropping and maize–soybean rotation. The experimental site was located at the Yanming Lake Seed Company base in Shaheyan, Guandian Town, Dunhua City, Yanbian Korean Autonomous Prefecture, Jilin Province, China (128.3592°E, 43.4400°N). The climate is temperate semi-humid, and the soil type is Albic Luvisol. The mean monthly rainfall and mean air temperature during the crop-growing seasons of 2012–2022 are shown in **Figure 1**. To investigate the long-term effects of crop rotation and continuous monoculture, sampling for this study was conducted from October 1, 2020, to October 1, 2022. Prior to sampling, all treatment groups had been implemented continuously for nine years. The maize cultivar used was Deyu 579, while the soybean cultivar was Jiyu 47. Fertilizers applied included urea (N_2_O ≥ 46%), superphosphate (P_2_O_5_ ≥ 46%), and potassium sulfate (K_2_O ≥ 52%). Fertilizer application rates were precisely calibrated manually according to experimental design. A one-time basal fertilization method was employed, with fertilizers applied simultaneously during sowing. From 2012 to 2022, the crop cultivars, fertilization methods, and fertilizer types remained consistent across experimental fields. Additionally, during this period, the rotation treatment maintained alternating maize-soybean cropping on the same plot, while the continuous monoculture treatment involved repeated cultivation of the same crop on identical plots. The basic physicochemical properties of soils under different treatments from 2020 to 2022 are detailed in **Supplementary Table S1** of the **Supplementary Materials**.

**Figure 2:**
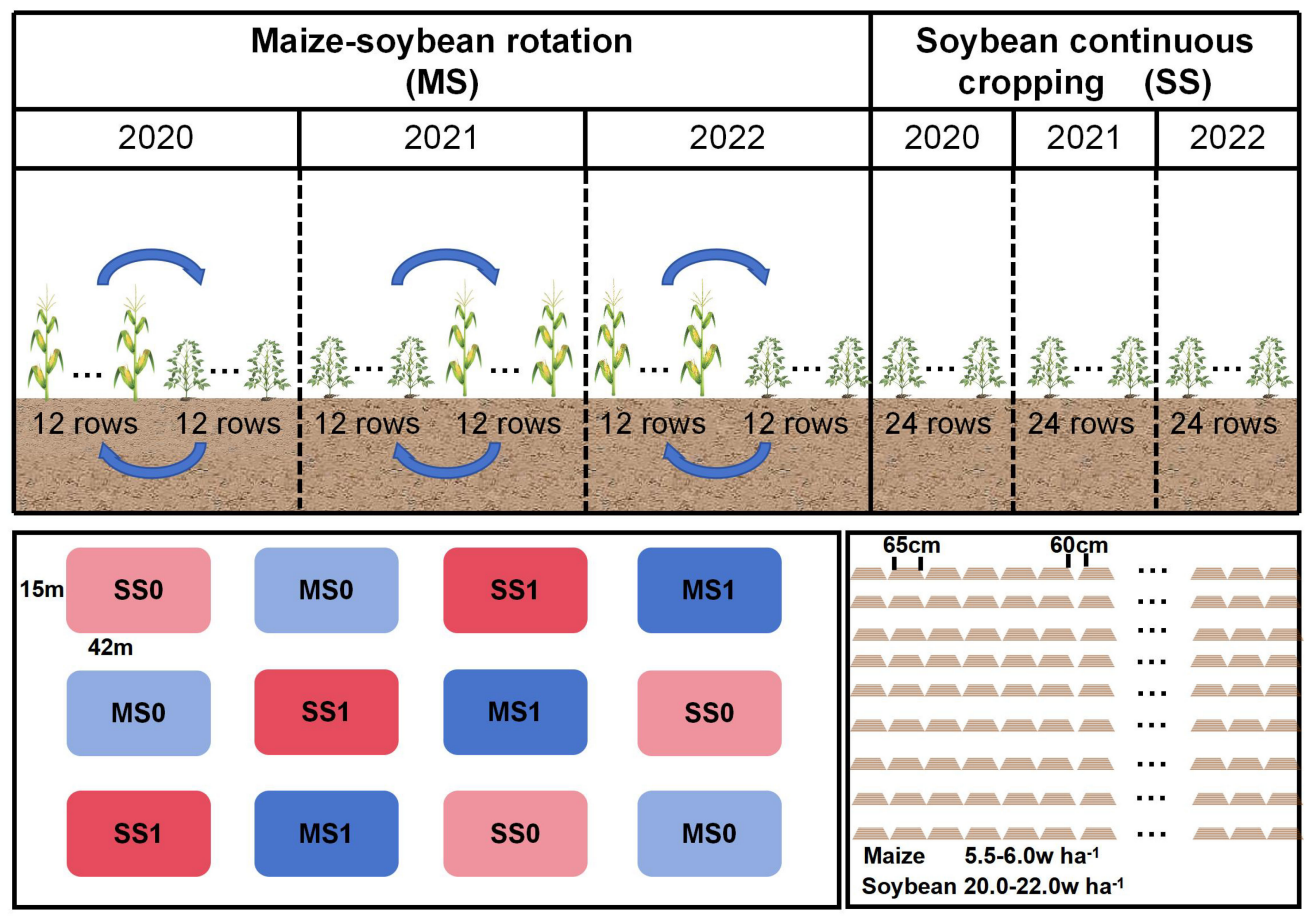
Planting distribution map. MS1: Fertilized soybean-maize rotation, MS0: Non-fertilized soybean-maize rotation, SS1: Fertilized continuous soybean cultivation, and SS0: Non-fertilized continuous soybean cultivation.

The study was carried out on twelve equal-sized plots. The experiment featured four treatment groups: MS1 - Fertilized soybean-maize rotation, MS0 - Non-fertilized soybean-maize rotation, SS1 - Fertilized continuous soybean cultivation, and SS0 - Non-fertilized continuous soybean cultivation. The treatments were replicated three times. Each experimental plot covered an area of 630 m^2^. The crops within each sub-plot were planted in 24 rows (MS1 or MS0: maize, 12 rows; soybean, 12 rows), with each row spanning 65 cm and a row spacing of 60 cm. **Figure 2** shows the planting distribution. The soybean planting density was 200,000 plants per hectare. The soybean population in the rotation trial field was 6,300, while that in the continuous monoculture trial field was 12,600 plants. The planting density of maize was 55,000 plants per hectare. The maize plant population in each trial field was ~1,733 plants. Two nitrogen (N) application levels were employed: 0 kg N ha^-1^ and 60 kg N ha^-1^. Additionally, phosphorus (P) and potassium (K) fertilizers were applied at rates of 75 kg P_2_O_5_ ha^-1^ and K_2_O ha^-1^, respectively. Pest and disease control, and intertillage weeding remained consistent each year.

### 2.2 Sample collection and analysis

For three consecutive years, soil samples were collected from the 0–20 cm plow layer of soybean fields during the soybean maturity stage, while no sampling was conducted in the maize fields. Three sampling points were established within each plot, using the soil augering method. Fresh soil specimens were extracted *in situ* from the 0–20 cm soil depth on the ridges. These were homogenized thoroughly before being placed in ice chests. Subsequently, extraneous elements such as stones and vegetative residues were removed. A portion of the soil samples was air-dried and reserved for chemical analysis, while another fraction, designated for the quantification of microbial biomass and enzymatic activity, was stored in a refrigerator at -80°C. Prior to harvest, the plant height, stem diameter, and node number of 10 consecutive soybean plants were measured. Additionally, three random sampling areas of 10 m² each were selected from each mature soybean field for manual harvesting. After threshing, the grain weight was measured and subsequently converted into yield per hectare based on the harvested area.

Soil total nitrogen (TN) was determined using the Kjeldahl method for digestion (Te et al., 2022), followed by filtration through a 0.45 μm PES membrane. A continuous flow analyzer (AA3, AutoAnalyzer 3, Technicon, Windows/NT) was used for analysis. Soil inorganic nitrogen (TIN), ammonium nitrogen (AN), and nitrate nitrogen (NN) were extracted with 2 mol L^-1^ CaCl_2_, shaken at 180 rpm for 60 min, allowed to settle for 30 min, and then subjected to AA3 analysis (Neels et al., 2024). Soil alkali hydrolyzable nitrogen (AAN) and acid hydrolyzable nitrogen (AHN) were determined using the alkaline diffusion method (Zhang et al., 2019; Hou et al., 2023). Total organic nitrogen (TON), light fraction organic nitrogen (LON), and heavy fraction organic nitrogen (RON) were quantified using the semi-micro Kjeldahl method (Dou et al., 2017).

The soil pH and electrical conductivity (EC) values were extracted from the soil-water mixture at a ratio of 5:1. The pH was subsequently measured using a pH meter (pH-100A, 100-2000rpm, LICHEN, Shanghai, CN), while the EC was determined using a conductivity meter (DDSJ-11A-307, YUEPING, Shanghai, CN). For the analysis of total potassium (TK) and total phosphorus (TP) components in the soil, a digestion method involving concentrated perchloric acid and sulfuric acid was employed. The digested solution was filtered through a 0.45 μm PES membrane, and the content was quantified using flame photometry (FP6400, INESA, Shanghai, CN) and the AA3 analyzer, respectively. The readily available phosphorus (AP) component in the soil was extracted using a sodium bicarbonate solution. The extraction process involved shaking at 180 rpm per minute for 2 h, followed by a 30 min settling period. After filtration through a 0.45 μm PES membrane, the concentration of AP was measured using the AA3 analyzer. Similarly, the available potassium (AK) component in the soil was extracted using an ammonium acetate solution, involving shaking at 180 rpm for 2 h, followed by a 30 min settling period. After filtration through a 0.45 μm PES membrane, the concentration of AK was determined using flame photometry (fp6400, INESA, Shanghai, CN). The soil’s organic matter content was determined using the potassium dichromate volumetric method combined with the dilution-heat approach (Zhang et al., 2024).

### 2.3 Determination of enzymatic activities related to soil nitrogen transformation

Soil urease (SU) activity was assayed using the urease colorimetric method with urea as the substrate. The soil sample was incubated in a C_6_H_8_O_7_ buffer solution (37°C,pH 6.7) for 24 h, and measured at 578 nm using a spectrophotometer (Lee et al., 2021). Soil protease (SP) activity was determined using the casein colorimetric method. The soil sample was incubated in a phosphate buffer solution (37°C, pH 5.5) for 24 h, and read at 650 nm using a spectrophotometer. Soil nitrate reductase (SNR) activity was assessed through anaerobic cultivation followed by the phenol-sulfuric acid colorimetric method. The soil sample was incubated in a glucose buffer solution (30°C, pH 7.0) for 24 h, followed by a spectrophotometric study at 400–500 nm (Deiglmayr et al., 2006).

### 2.4 PCR amplification and high-throughput sequencing

Total genomic DNA from soil microbial communities was extracted according to the instructions of the E.Z.N.A.^®^ Soil DNA Kit (Omega Bio-tek, Norcross, GA, U.S.). The quality of the extracted genomic DNA was assessed using 1% agarose gel electrophoresis, while DNA concentration and purity were determined using a NanoDrop 2000 spectrophotometer (Thermo Scientific, U.S.). For the amplification of bacterial 16S rRNA gene fragments corresponding to the V3-V4 region, the primers 338F (5’-ACTCCTACGGGAGGCAGCAG-3’) and 806R (5’-GGACTACHVGGGTWTCTAAT-3’) were employed. In the case of fungal 18S rRNA gene amplification targeting the ITS1 region, the primers ITS1F (5’-CTTGGTCATTTAGAGGAAGTAA-3’) and ITS2 (5’-GCTGCGTTTCTTTCATCGATGC-3’) were utilized. Following the amplification, the PCR products were purified, quantified, and standardized to generate sequencing libraries. The 16S rRNA gene libraries were prepared as paired-end (PE) 2 × 300, while the 18S rRNA gene libraries were prepared as PE 2 × 250. The constructed libraries were subjected to rigorous quality control. Subsequently, the qualified libraries were subjected to sequencing on the Illumina NovaSeq 6000 platform (Hou et al., 2023). The registration number for this biological project is MJ20221021156-MJ-M-20221024089.

### 2.5 Statistical analysis

Statistical analysis was performed using SPSS 22.0 software. The effects of planting patterns and fertilization on soybean agronomic traits, yield, soil chemical properties, and enzyme activities were evaluated using two-way analysis of variance (ANOVA). To assess the primary effects of fertilization, growth stages, and their interactions on soil nitrogen forms and enzymatic activity, a bidirectional analysis of variance was employed. Pearson correlation tests were carried out to evaluate the relationships between microbial modules, enzymes, relative abundances of nitrogen forms, and the physicochemical properties of the soil along with enzyme activity.

Beta diversity analysis based on the Bray-Curtis dissimilarity coefficient and PCA analysis was used to compare the similarity of species community diversity among different samples. A co-occurrence network model at the genus level was established for continuous and rotational soil microbial communities, comparing the interactions between soil microbial communities under different cultivation modes.

Visual analysis of microbial ecological networks and topological indices were performed using Gephi software (version 0.9.6). The following topological indices were used to describe the nodes and connecting lines in the network 1) the number of connecting lines of a node, which is the number of all connecting lines connected to each node; 2) the median centrality of a node, which is the node located on the shortest path between two nodes, calculated as in the Equation 1; 3) the topological coefficient of a node, which embodies the proximity of the nodes and is expressed by the Equation 2; 4) the connecting line weights, which reflect the number of connections between a particular OTU node and the sample node; 5) the connecting line centrality, a parameter that shows the importance of each connecting line in the information transfer process of the whole network.


(1) $$Cb(n)=\sum_{s\neq n\neq t}(\frac{\sigma_{st}(n)}{\sigma_{st}})$$

Where n is the destination node, s and t are nodes in the network other than n, ${\text{σ}}_{\text{st}}$ represents the number of shortest paths from node s to node t, and ${\text{σ}}_{\text{st}}(\text{n})$ denotes the number of shortest paths from node s to node t that must pass through node n.


(2) $$T_{n}\;=\;\frac{avg(J_{\left(n,m\right)})}{k_{n}}$$

Where ${\text{J}}_{\left(\text{n},\text{m}\right)}$ is the number of all nodes adjacent to both nodes n and m, and the value of ${\text{J}}_{\left(\text{n},\text{m}\right)}$ is increased by 1 if n is directly adjacent to m. ${\text{k}}_{\text{n}}$ is the number of all neighbors of the node.

Structural equation modeling (SEM) analysis was conducted using the dplyr, linkET, ggplot2, and plspm packages in R version 4.3.1 (https://www.r-project.org/) to explore the direct or indirect effects of crop rotation and nitrogen fertilization on soil microbial communities. Three indices—Comparative Fit Index (CFI), Root Mean Square Error of Approximation (RMSEA), and Standardized Root Mean Square Residual (SRMR)—were selected to evaluate the goodness-of-fit of the structural equation model.

## 3 Results

### 3.1 Agronomic traits and yield of soybean

Different cropping patterns and nitrogen fertilizer inputs exerted significant impacts on soybean stem diameter, plant height, number of nodes, grain weight per plant, 100-grain weight, and overall yield (**Table 1**). During 2020-2022, under continuous cropping treatments, all measured indicators consistently demonstrated SS1 > SS0, with an average increase of 25.76%, 18.39%, 5.54%, 54.09%, 8.89%, and 28.73%, respectively. Similarly, in rotation cropping treatments over the same period, all indicators exhibited an upward trend, consistently showing MS1 > MS0, with an average increase of 15.5%, 11.91%, 9.51%, 28.23%, 6.77%, and 22.74%, respectively. Compared to the SS0 treatment, the MS0 treatment resulted in an average reduction of 1.65% in the number of nodes, while other indicators rose by 11.06%, 10.12%, 23.22%, 3.27%, and 12.08%, respectively. The stem diameter, plant height, 100-grain weight, and yield exhibited a decreasing trend with increasing years of continuous cropping. Contrarily, in rotation treatments, the number of nodes, 100-grain weight, and yield demonstrated an increasing trend. Two-way ANOVA revealed that both cropping pattern and nitrogen fertilizer input significantly correlated with stem diameter, plant height, grain weight per plant, and yield. Fertilization emerged as the primary factor impacting soybean yield, with long-term continuous cropping exerting detrimental effects. Comparisons between MS0 or SM0 treatments and SS0 treatments indicated that rotation effectively enhanced soybean yield, while also improving agronomic traits and increasing yield components.

**Table 1:** Changes in agronomic traits and yield of soybean under different treatments from 2020 to 2022 (n=30).

Years	Treatment	Stem diameter (mm)	Height (cm)	Number of nodes	Total grain weight (g)	100-grain weight (g)	Yield (kg ha^-1^)
2020	MS1	7.38 ± 0.56 ab	87.63 ± 4.67 a	15.00 ± 0.15 b	156.36 ± 8.69 a	18.63 ± 1.01 ab	2894.44 ± 82.21a
	MS0	6.90 ± 0.44 c	75.99 ± 5.12 c	14.00 ± 1.25 c	114.95 ± 6.90 c	17.13 ± 1.01 c	2380.11 ± 64.38 c
	SS1	7.85 ± 0.34 a	83.32 ± 2.98 b	15.00 ± 0.35 b	152.19 ± 2.40 a	18.81 ± 0.52 a	2777.78 ± 107.15b
	SS0	6.49 ± 0.59 d	63.55 ± 4.09 d	15.00 ± 0.46 a	114.86 ± 6.09 c	16.11 ± 3.39 d	2150.00 ± 180.28d
2021	MS1	7.89 ± 0.13 a	84.49 ± 0.93 a	16.00 ± 0.25 a	157.13 ± 8.67 a	18.67 ± 1.27 a	2912.67 ± 146.71a
	MS0	6.50 ± 0.53 d	76.45 ± 5.96 c	15.00 ± 0.57 bc	122.80 ± 6.96 d	17.50 ± 0.40 cd	2381.67 ± 154.95d
	SS1	7.47 ± 0.53 b	82.03 ± 2.37 b	15.00 ± 0.89 b	153.77 ± 3.17 b	18.20 ± 1.14 b	2737.33 ± 6.66 b
	SS0	5.80 ± 0.47 e	73.02 ± 1.63 c	14.00 ± 1.03 c	93.23 ± 4.67 e	17.37 ± 0.35 d	2129.00 ± 93.92 d
2022	MS1	7.83 ± 0.52 a	84.58 ± 9.38 a	17.00 ± 1.25 a	150.69 ± 7.24 a	18.68 ± 1.19 a	2983.33 ± 236.29a
	MS0	6.63 ± 0.13 d	76.97 ± 1.18 c	15.00 ± 0.31 d	124.83 ± 1.21 c	17.62 ± 0.39 c	2400.00 ± 132.29c
	SS1	7.35 ± 0.66 b	81.03 ± 2.92 b	16.00 ± 0.72 b	149.25 ± 5.74 a	18.04 ± 1.05 b	2711.07 ± 19.25 b
	SS0	5.77 ± 0.28 e	72.55 ± 1.63 d	14.00 ± 1.02 d	90.54 ± 9.05 d	17.16 ± 0.31 d	2111.11 ± 67.36 d
Two-factor analysis of variance (F-value)							
C		17.09**	29.57**	1.11	29.97**	8.99*	422.19**
N		29.06**	22.17**	4.26*	50.26**	17.71**	489.83**
C×N		10.59**	9.79*	0.05	22.29**	3.44	97.57**

Statistically significant differences (*P* < 0.05) between the two treatments are denoted by distinct letters (a, b, c). MS1: Fertilized soybean-maize rotation, MS0: Non-fertilized soybean-maize rotation, SS1: Fertilized continuous soybean cultivation, and SS0: Non-fertilized continuous soybean cultivation. C: cropping pattern, N: Nitrogen fertilizer input. * represents *P* < 0.05, ** represents *P* < 0.01.

### 3.2 Fundamental physicochemical characteristics of soil

From 2020 to 2022, except for TP, crop rotation regimen outcomes differed significantly (*P* < 0.05) from those of the SS1 and SS0 treatments (**Table 2**). When compared to continuous monoculture, soil pH and SOM demonstrated augmentation in the crop rotation systems, exhibiting increments of 0.48% to 14.31% and 20.33% to 77.36%, respectively. Conversely, EC, TK, AP, and AK constituents experienced a reduction. It is noteworthy that despite the amplified soil pH and SOM under crop rotation practices, the introduction of fertilizers interrupted this growth trend. Furthermore, increased fertilizer application exacerbated the inhibitory impact. Within the continuous monoculture approach, soil EC, AP, and AK showed a propensity for post-fertilization augmentation. The two-way ANOVA reflected that cropping pattern, and nitrogen fertilizer input exhibited highly significant correlations with EC, SOM, AP, and AK. Their interactions also demonstrated highly significant correlations with EC, SOM, and AP (*P* < 0.01), and a significant correlation with TP (*P* < 0.05). Overall, nitrogen-based fertilizers application led to a decline in soil pH, SOM, TP, and TK, regardless of the duration of the crop rotation cycle, while concurrently fostering a rise in EC, AP, and AK.

**Table 2:** Changes in soil physicochemical parameters under different treatments from 2020 to 2022 (n=3).

Years	Treatment	pH	EC	SOM	TP	TK	AP	AK
			(μS cm^-1^)	(g kg^-1^)	(g kg^-1^)	(g kg^-1^)	(mg kg^-1^)	(mg kg^-1^)
2020	MS1	6.76 ± 0.31 b	40.29 ± 3.00 a	37.25 ± 0.33 b	0.82 ± 0.11 c	6.28 ± 0.02 c	37.54 ± 0.85 b	55.06 ± 1.79 c
	MS0	6.94 ± 0.02 a	13.98 ± 0.35 c	46.23 ± 3.39 a	1.05 ± 0.14 a	6.98 ± 0.01 b	15.36 ± 0.38 c	44.25 ± 1.67 d
	SS1	6.29 ± 0.06 d	42.98 ± 4.92 a	28.16 ± 1.14 c	1.00 ± 0.05 b	7.20 ± 0.02 a	83.45 ± 0.93 a	97.01 ± 1.59 a
	SS0	6.44 ± 0.03 c	12.28 ± 1.22 c	26.64 ± 0.81 c	0.98 ± 0.01 b	7.18 ± 0.07 a	19.61 ± 1.43 c	76.29 ± 0.23 b
2021	MS1	6.78 ± 0.08 b	39.73 ± 1.60 a	33.44 ± 5.12 c	0.93 ± 0.11 c	6.44 ± 0.02 c	48.73 ± 1.85 b	56.37 ± 3.07 c
	MS0	7.03 ± 0.03 a	17.57 ± 1.38 c	40.30 ± 6.73 a	1.07 ± 0.05 a	6.71 ± 0.08 b	17.37 ± 0.59 d	35.53 ± 3.40 d
	SS1	6.28 ± 0.06 d	42.77 ± 4.92 a	27.95 ± 1.14 d	0.99 ± 0.05 b	6.99 ± 0.02 a	73.56 ± 0.42 a	103.80 ± 2.31 a
	SS0	6.52 ± 0.03 c	14.32 ± 0.92 c	29.19 ± 0.69 d	0.96 ± 0.01 b	7.06 ± 0.07 a	17.15 ± 0.73 d	82.83 ± 3.01 b
2022	MS1	6.18 ± 0.01 c	32.93 ± 3.36 b	35.19 ± 6.06 b	1.03 ± 0.01 b	6.53 ± 0.06 bc	40.55 ± 1.82 b	61.67 ± 0.47 b
	MS0	6.86 ± 0.03 a	12.53 ± 0.80 c	47.25 ± 3.98 a	1.17 ± 0.06 a	6.41 ± 0.17 c	19.67 ± 2.71 c	34.23 ± 0.67 c
	SS1	6.15 ± 0.03 c	51.17 ± 2.17 a	36.44 ± 1.49 c	1.02 ± 0.02 b	6.93 ± 0.03 ab	72.62 ± 0.88 a	93.66 ± 5.40 a
	SS0	6.34 ± 0.13 b	17.28 ± 4.16 c	39.45 ± 1.21 c	1.05 ± 0.01 ab	7.09 ± 0.03 a	12.63 ± 0.93 d	91.93 ± 0.38 a
Two-factor analysis of variance (F-value)								
C		1.79	8.79**	255.58**	2.37	13.57**	141.99**	87.93**
N		0.32	408.53**	28.42**	0.69	2.71	924.88**	14.47**
C×N		0.03	9.92**	23.54**	4.21*	3.29	159.71**	0.22

Statistically significant differences (*P* < 0.05) between the two treatments are denoted by distinct letters (a, b, c). SOM, organic matter; TP, total phosphorus; TK, total potassium; AP, available phosphorus; AK, available potassium. MS1: Fertilized soybean-maize rotation, MS0: Non-fertilized soybean-maize rotation, SS1: Fertilized continuous soybean cultivation, and SS0: Non-fertilized continuous soybean cultivation. C: cropping pattern, N: Nitrogen fertilizer input. * represents *P* < 0.05, ** represents *P* < 0.01.

### 3.3 Transformation of various nitrogen forms in soil

**Figure 3:**
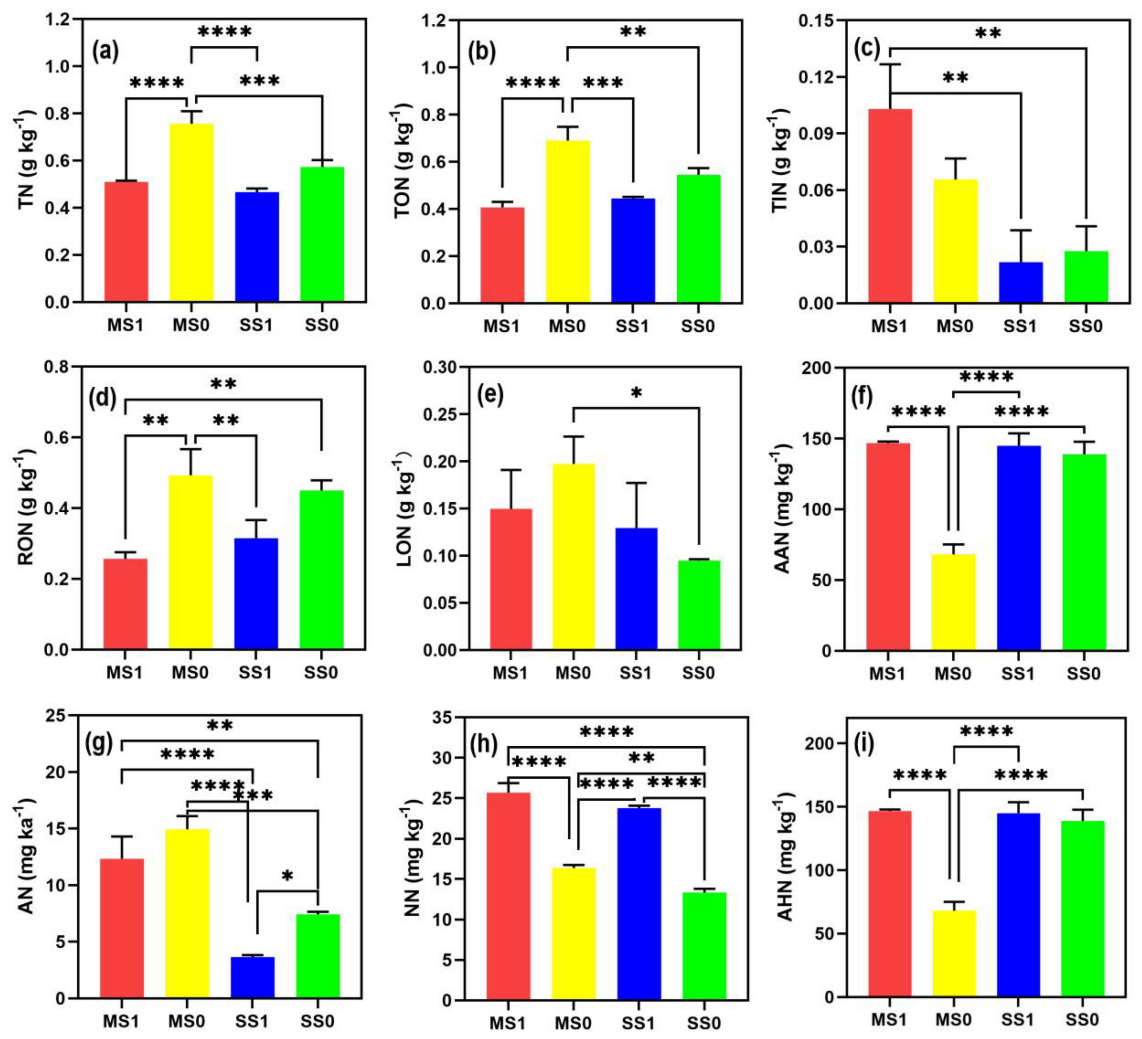
Variations in different nitrogen forms (n=9). For each treatment, We fitted the data for 2020-2022, and for consistency across treatments, the two treatments in the rotation are represented equally by MS1 and MS0. The standard deviations of the three replicates are represented by error bars, while asterisk denote significant differences between treatments (*P < 0.05; **P < 0.01; ***P < 0.001; ****P < 0.0001). **(A)** TN, total nitrogen; **(B)** TON, total organic nitrogen; **(C)** TIN, total inorganic nitrogen; **(D)** RON, heavy fraction organic nitrogen; **(E)** LON, light fraction organic nitrogen; **(F)** AAN, alkali hydrolyzable nitrogen; **(G)** AHN, acid hydrolyzable nitrogen; **(H)** AN, ammonium nitrogen; and **(I)** NN, nitrate nitrogen. MS1: Fertilized soybean-maize rotation, MS0: Non-fertilized soybean-maize rotation, SS1: Fertilized continuous soybean cultivation, and SS0: Non-fertilized continuous soybean cultivation.

**Figure 3** depicts that crop rotation increases soil TN content as compared to continuous cropping, with the effect being more pronounced on the increase in TIN content. In the comparison between MS1 and SS0 treatments, MS1 exhibited a substantial rise of 273.43% in TIN content. In the crop rotation treatments, as nitrogen application rates increased, both soil TN and TON content gradually rose, while TIN content progressively declined. Crop rotation also enhances the content of LON. Specifically, the MS0 treatment presented the highest LON content, outperforming MS1 and SS0 by 31.98% and 108.34% respectively. Among the crop rotation treatments, MS1 accounted for 36.79% of the LON proportion, while MS0 and SS0 constituted 28.60% and 17.30% of the total, respectively. MS1 and SS1 treatments had significantly higher AAN content than the others. This implied a close relationship between soil AAN content and nitrogen application rates. Across all treatments, AHN, NN, and AN constituted 68.53%-87.04%, 8.35%-16.47%, and 2.16%-15.03% of AAN, respectively. Notably, crop rotation resulted in an 11.45% lower proportion of AHN compared to continuous cropping. Contrarily, NN and AN proportions were higher by 3.87% and 7.58%, respectively.

Reduced nitrogen application rates across all treatments corresponded to an increase in AHN proportion and a decrease in NN and AN proportions. From the aforementioned analysis, it is evident that compared to SS0, crop rotation significantly enhanced the content of LIN, LON, AAN, NN, and AN in the soil. Furthermore, with an increase in the number of years of crop rotation, these components displayed an ascending trend. During the soybean maturation stage, nitrogen fertilizer application notably augmented AAN content. It is worth noting that both nitrogen application rates and years of cultivation exhibited interactive effects on the transformation of soil nitrogen forms.

### 3.4 Changes in key enzyme activities during the nitrogen conversion process

The activities of three enzymes pertinent to the nitrogen cycle exhibited pronounced disparities between distinct cultivation methodologies with or without nitrogen fertilization treatments (**Table 3**). In comparison to the N0 treatment, both SU and SP activities were elevated by 3.95% to 29.64% and 38.82% to 76.83%, respectively, under the rotational cropping regimen. Meanwhile, the SNR of the rotational cropping regimen exhibited a reduction of 17.41% to 51.86% in relation to the SS0 treatment. Concurrent with the augmentation of nitrogen levels, analogous patterns of enzymatic activity alterations were observed for both rotational and continuous cropping approaches. In the former, the activities of SU and SNR rose, with increments of 19.79% and 41.72% respectively in MS1 compared to MS0, and increments of 4.07% and 23.56% respectively in SS1 compared to SS0. Conversely, the activity of SP declined, with reductions of 21.50% in MS1 compared to MS0 and 7.84% in SS1 compared to SS0. Comparing the years 2020 and 2022, rotational cropping showcased an incremental and steady rise in SR and SP activities, alongside a reduction in SNR activity. In contrast, the continuous cropping approach demonstrated an opposing trend in the alteration of SU, SP, and SNR activities. Notably, the interplay between nitrogen application rates and years of cultivation exerts a mutual influence on key soil enzymatic activities across all treatments. The two-way ANOVA showed that both cropping pattern and nitrogen fertilizer input exhibited highly significant correlations with the activities of all three enzymes. Albeitinteractions, a highly significant correlation was observed with SU (*P* < 0.01), a significant correlation with SP (*P* < 0.05), but no significant correlation with SNR (*P* > 0.05).

**Table 3:** Activities of key enzymes involved in nitrogen transformation processes during 2020-2022 (n=3).

Years	Treatment	SU	SP	SNR
		mg g^-1^ d^-1^	mg g^-1^ d^-1^	μm g^-1^ d^-1^
2020	MS1	4.32 ± 0.08 a	54.14 ± 3.18 b	9.18 ± 0.18 c
	MS0	3.57 ± 0.05 bc	64.23 ± 3.78 a	6.23 ± 0.08 d
	SS1	3.65 ± 0.04 bc	29.65 ± 0.24 c	13.71 ± 0.47 a
	SS0	3.49 ± 0.02 c	32.84 ± 0.96 c	11.17 ± 0.67 b
2021	MS1	4.53 ± 0.08 a	55.68 ± 1.08 b	6.56 ± 0.06 bc
	MS0	3.59 ± 0.06 b	70.94 ± 0.87 a	3.21 ± 0.03 d
	SS1	3.61 ± 0.02 b	33.68 ± 0.09 c	10.46 ± 0.32 a
	SS0	3.46 ± 0.04 b	37.89 ± 1.01 c	8.06 ± 0.07 b
2022	MS1	4.59 ± 0.08 a	43.24 ± 1.44 b	5.81 ± 0.02 b
	MS0	3.62 ± 0.05 b	59.80 ± 0.96 a	3.12 ± 0.05 c
	SS1	3.55 ± 0.05 b	38.29 ± 0.07 b	9.96 ± 0.11 a
	SS0	3.42 ± 0.08 b	39.53 ± 0.74 b	6.86 ± 0.05 b
Two-factor analysis of variance (F-value)				
C		494.25**	207.88**	58.05**
N		507.06**	28.65**	24.68**
C×N		222.22**	12.41*	0.08

SU: urease: SP: protease; and SNR: nitrate reductase. MS1: Fertilized soybean-maize rotation, MS0: Non-fertilized soybean-maize rotation, SS1: Fertilized continuous soybean cultivation, and SS0: Non-fertilized continuous soybean cultivation. C: cropping pattern, N: Nitrogen fertilizer input. * represents *P* < 0.05, ** represents *P* < 0.01.

### 3.5 Changes in soil microbial communities

#### 3.5.1 Composition of the bacterial and fungal communities

**Figure 4:**
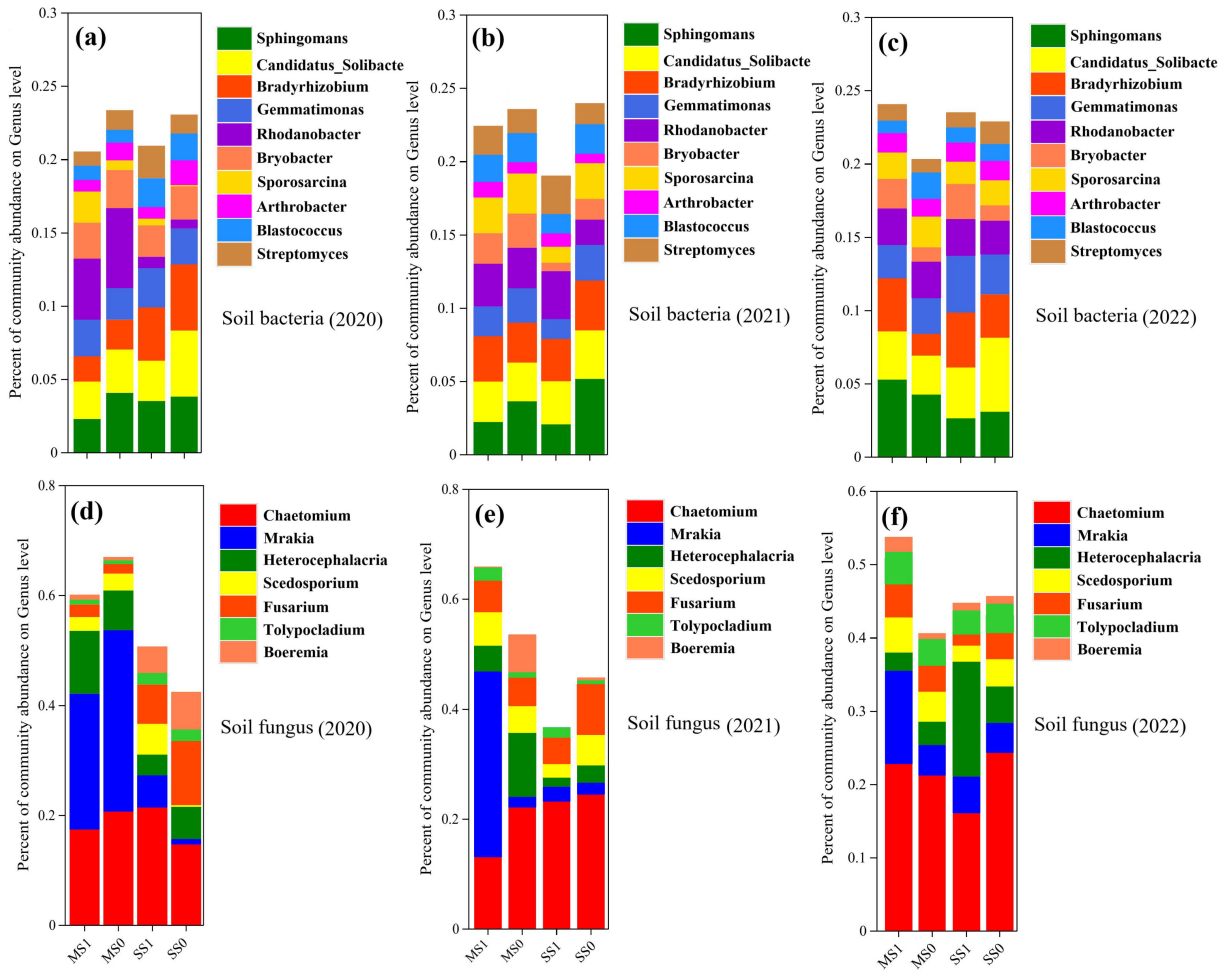
A comparative assessment of the relative abundance of microbial genus in soil, in light of divergent agronomic systems (n=3) during the years 2020 to 2022. **(A-C)** represent soil bacterial communities from 2020 to 2022, while **(D-F)** depict soil fungal communities. MS1: Fertilized soybean-maize rotation, MS0: Non-fertilized soybean-maize rotation, SS1: Fertilized continuous soybean cultivation, and SS0: Non-fertilized continuous soybean cultivation.

**Figure 4** shows the histograms of the horizontal distribution of the detected microbial communities under different treatments (). Among them, *Sphingomonas*, *Candidatus_Solibacter*, *Bradyrhizobium*, *Gemmatimonas*, *Rhodanobacter* and *Bryobacter* were the dominant genera in bacteria. Their total relative abundance accounted for 14.54%-19.49% of the total. Among fungi, *Chaetomium*, *Mrakia*, *Heterocephalacria*, *Scedosporium* and *Fusarium* were the dominant genera with a total relative abundance of 41.39-64.83%.

The relative abundance of Sphingomonas showed a decreasing trend with increasing planting years in the continuous treatments (SS1 and SS0), decreasing by an average of 14.19% up to 2022; *Candidatus_Solibacter, Bradyrhizobium, Gemmatimonas, Rhodanobacter* and *Bryobacter* (**Figures 4A-C**). gradually increased in relative abundance with an average of 25.57%, 3.39%, 25.88%, 129.35% and 55.96% up to 2022. The changes in bacterial dominant genera in the rotational treatments (MS1, MS0) were different from those in the continuous treatments. *Candidatus_Solibacter*, *Rhodanobacter* and *Bryobacter* were reduced by an average of 19.56, 47.3 and 53.85%, respectively. *Sphingomonas*, *Bradyrhizobium* and *Gemmatimonas* increased their relative abundance by 21.13%, 22.46% and 2.68%, respectively. Bacterial genera increased by 3.5%, 42.23%, 20.41%, 31.55% and 12.08% in the post-rotation fertilizer treatments except *Candidatus_Solibacter*, which decreased by 24.82%.

The relative abundance of *Chaetomium*, *Scedosporium* and *Fusarium* in the continuous treatments showed an incremental trend with increasing planting years (**Figures 4D-F**), with an average increase of 14.55%, 11.71% and 47.52% by 2022, while the relative abundance of *Mrakia*, *Heterocephalacria* showed a gradual decrease in relative abundance, with an average decrease of 11.24%. The changes in the dominant fungal genera in the rotational treatments (MS1 and MS0) were different from those in the continuous treatments, in which the relative abundance of *Mrakia* showed a gradual increase, with an average increase of 33.26% by 2022, and the relative abundance of *Chaetomium*, *Heterocephalacria*, *Scedosporium* and *Fusarium* showed a gradual decrease, with an average decrease of 62.47% by 2022. 2022 with an average decrease of 62.47%, 11.53%, 14.86% and 5.68% respectively. *Chaetomium* and *Fusarium* decreased by 32.49% and 25.63% respectively, while the remaining genera increased by 6.82%, 20.63% and 3.37% respectively, after crop rotation.

From the above analyses, it is clear that rotation was effective in increasing the relative abundance of *Gemmatimonas*, *Rhodanobacter* and *Mraki*a compared to the continuous treatment. Rotation suppressed the accumulation of *Bradyrhizobium*, *Chaetomium*, *Fusarium*, and fertilization was more effective in suppressing these two fungal genera.

#### 3.5.2 PCA analysis of bacteria and fungi

**Figure 5:**
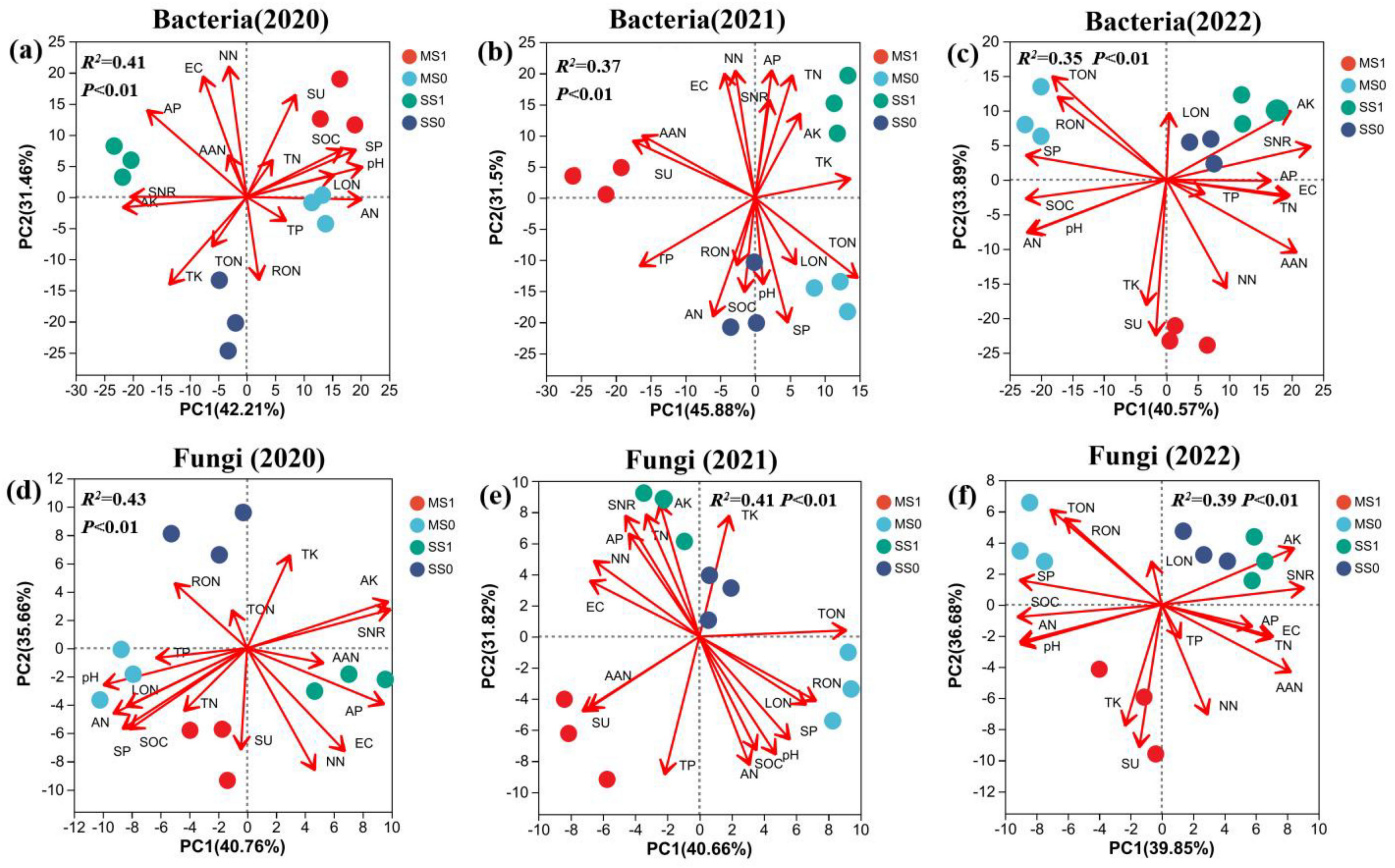
The PCA plot illustrating the Bray-Curtis distances based on OTUs (operational taxonomic units) of bacterial **(A-C)** and fungal **(D-F)** communities in soil across four experimental groups during the 2020–2022 period. The plot’s uppercase letters and red arrows represent environmental factors. MS1: Fertilized soybean-maize rotation, MS0: Non-fertilized soybean-maize rotation, SS1: Fertilized continuous soybean cultivation, and SS0: Non-fertilized continuous soybean cultivation. SOM, organic matter; TP, total phosphorus; TK, total potassium; AP, available phosphorus; AK, available potassium; TN, total nitrogen; TON, total organic nitrogen; TIN, total inorganic nitrogen; RON, heavy fraction organic nitrogen; LON, light fraction organic nitrogen; AAN, alkali hydrolyzable nitrogen; AHN, acid hydrolyzable nitrogen; AN, ammonium nitrogen; NN, nitrate nitrogen. SU: urease: SP: protease; and SNR: nitrate reductase.

During2020- 2022, at the bacterial phylum level (97% similarity), PCA revealed an average explanatory rate of 42.89% for the firstPC1) and 32.28% for the second PC2 (**Figure 5**; **Supplementary Table S2**). In the crop rotation treatments, namely MS0, MS1, SS1, and SS0, clear differentiation was observed for both PC1 and PC2. AAN, TON, RON, and NN displayed significant contributions to PC1, with rates of 82.16%, 52.95%, 51.32%, and 42.46%, respectively. AN and AK demonstrated notable contributions to the PC2, with rates of 77.64% and 61.92% respectively. SU and SP were aligned with the direction of MS1 treatments, highlighting the substantial influence of their magnitudes on the soil bacterial community in these two treatments.

Turning attention to the fungal community analysis under varying treatments, for the years 2020 through 2022, the Beta diversity analysis demonstrated an average explanatory rate of 40.42% for the PC1 and 34.65% for the PC2. As seen in bacterial communities, the varying treatments viz. MS0, MS1, SS1, and SS0 exhibited substantial differences in fungal populations. Notably, ANN and NN made notable contributions to the PC1, with rates of 65.26% and 51.92% respectively, while AK demonstrated a significant contribution of 71.62% to the PC2. Soil attributes SU, SP, and SNR did not significantly impact the variations in fungal communities across the crop rotation treatments.

#### 3.5.3 Co-occurrence networks and ecological assemblages of bacteria and fungi

**Figure 6:**
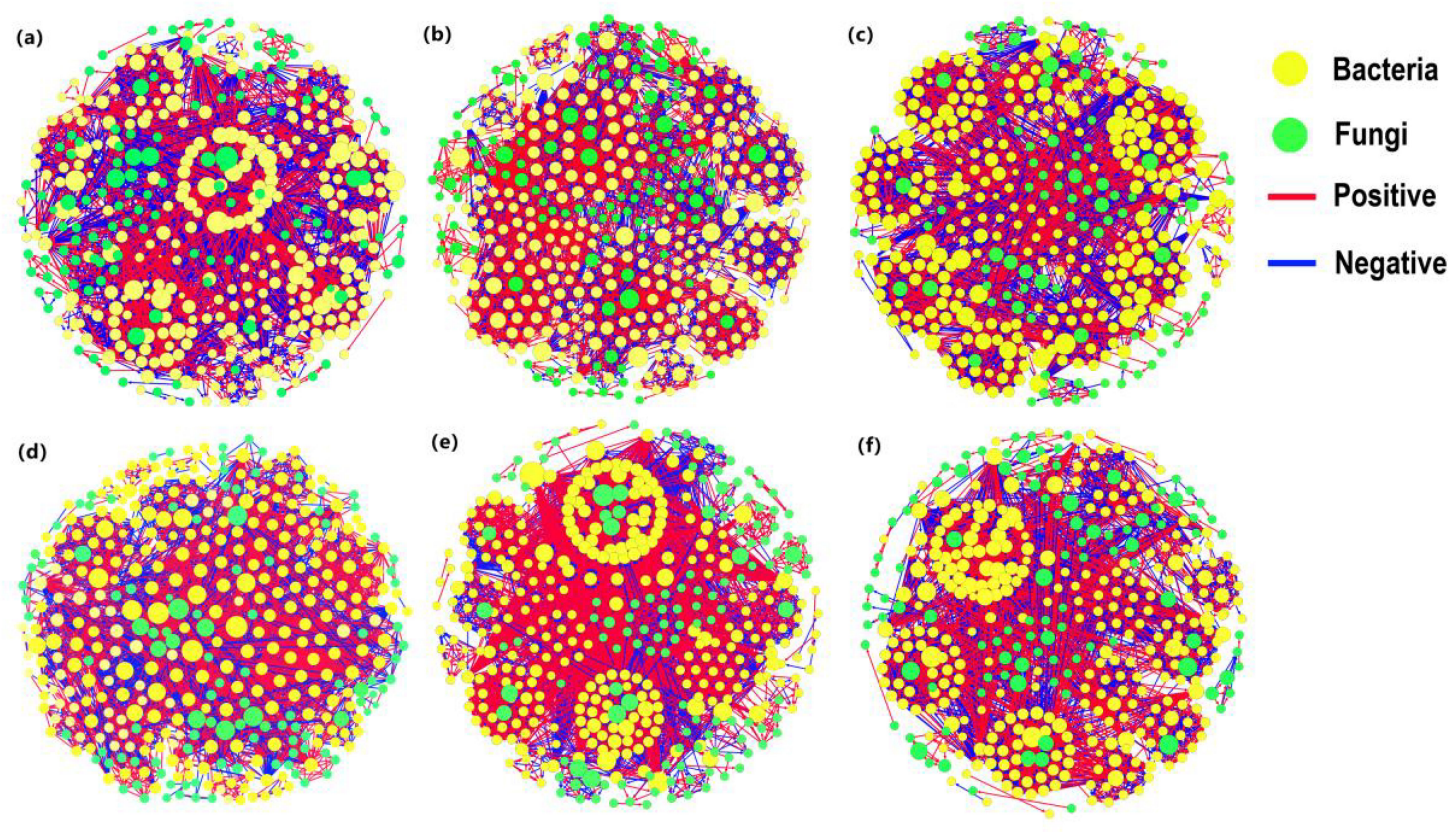
Co-occurrence networks of soil microbial communities, sculpted by the exquisite dance of correlation. **(A-C)** represent soil bacterial communities, while **(D-F)** depict soil fungal communities from 2020 to 2022. Within this intricate tapestry, each circle symbolizes a distinct biological entity, an embodiment of species diversity. The dimensions of these circles represent the tapestry’s complex weaving of species’ relative abundances. Interwoven between these circlets, filaments of connection manifest as slender bridges, indicating intricate relationships between pairs of organisms. The girth of these filaments signifies their interdependence.

This compilation was used to elucidate the interrelations among soil microbial communities under varying cultivation practices (**Figure 6**; **Table 4**). Crop rotation resulted in a reduction in soil microbial nodes as well as an increase in edge numbers. This implied reduced microbial diversity in crop-associated soil following crop rotation. However, interrelationships among different microbial phyla are complex. A comparison of microbial proportions between continuous cropping and crop rotation for the same year revealed that bacterial prevalence exceeded fungal prevalence in all treatments. Notably, crop rotation increased the proportion of bacteria, with a magnitude ranging between 1.75% and 9.86%. The positive correlations outweighed negative correlations in all treatments, with an average proportion of 55.15% and 44.85%, respectively. The crop rotation induced an augmentation in the proportion of positive correlations, with an increase ranging from 0.56% to 3.48%. The crop rotation treatments outperformed continuous cropping treatments in terms of average degree, average weighted degree, average clustering coefficient, and modularity. This pattern indicated that the interconnectivity among network nodes was stronger within the crop rotation treatments, featuring a more intricate and abundant web of connections. Nevertheless, both crop rotation and continuous cropping treatments showed an upward increase in node numbers, fungal proportions, edge numbers, negative correlation proportions, average degree, average weighted degree, and average clustering coefficient as planting years progressed.

**Table 4:** Topological property index of soil microbial co-occurrence networks.

Treatment		Node	Bacteria (%)	Fungus (%)	Edge	Positive (%)	Negative (%)	Average degree	Average weighting	Cluster coefficient	Modularity
2020	SS	414	70.62	29.38	3333	53.17	46.83	640.88	160.22	0.53	4.15
	MS	409	72.37	27.63	3562	55.44	44.56	652.59	163.15	0.56	4.29
2021	SS	426	69.01	30.99	3679	55.56	44.44	758.39	189.6	0.61	4.77
	MS	424	78.87	21.13	4121	56.12	43.88	764.22	191.06	0.64	4.92
2022	SS	440	57.59	42.41	3661	53.56	46.44	814.37	253.59	0.78	5.84
	MS	437	59.79	40.21	4317	57.04	42.96	817.42	254.36	0.81	5.99

Continuous soybean cropping (SS) versus soybean-maize rotation (MS). MS, soybean-maize rotation; SS, continuous soybean cultivation.

#### 3.5.4 Correlation analysis between soil microbial communities and soil environmental factors

**Figure 7:**
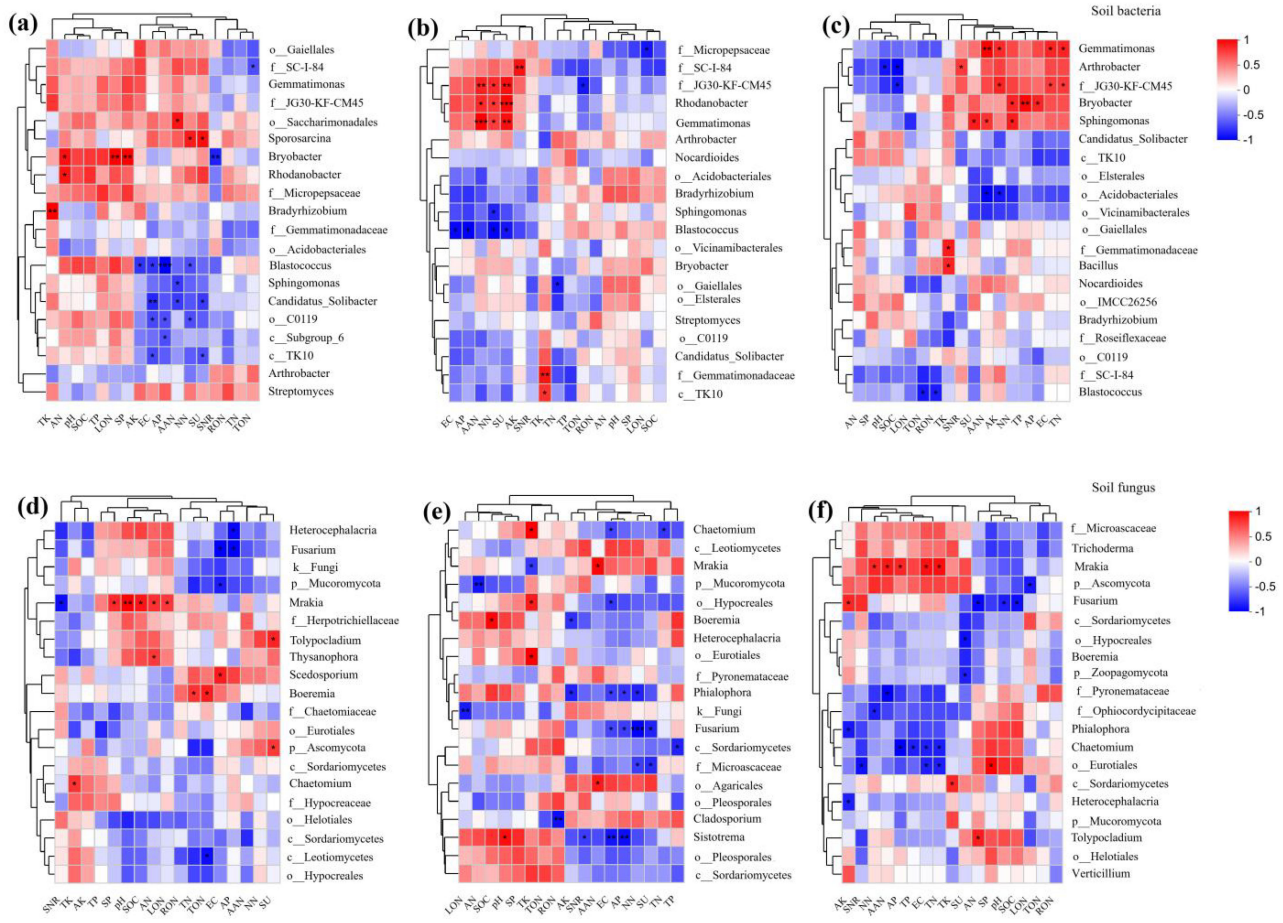
Heat map of genus-level diversity of soil microbial communities in relation to environmental factors, red represents positive correlation, blue represents negative correlation; * represents *P* < 0.05, ** represents *P* < 0.01, *** represents *P* < 0.001. **(A-C)** represent soil bacterial communities while **(D-F)** depict soil fungal communities from 2020 to 2022. SOM, organic matter; TP, total phosphorus; TK, total potassium; AP, available phosphorus; AK, available potassium; TN, total nitrogen; TON, total organic nitrogen; TIN, total inorganic nitrogen; RON, heavy fraction organic nitrogen; LON, light fraction organic nitrogen; AAN, alkali hydrolyzable nitrogen; AHN, acid hydrolyzable nitrogen; AN, ammonium nitrogen; NN, nitrate nitrogen. SU: urease, SP: protease, and SNR: nitrate reductase.

**Figure 7** shows the correlation thermograms of genus-level diversity of soil microbial communities in each treatment. It revealed that *Arthrobacter* was significantly negatively correlated with soil pH. *Candidatus_Solibacter* was highly significantly negatively correlated with soil EC.*Bryobacter* was positively correlated with AN and significantly positively correlated with LON.*Gemmatimonas* and *Rhodanobacter* showed a highly significant positive correlation with AAN and a significant positive correlation with NN. *Sporosarcina* also promoted the increase of soil AAN, but *Sphingomonas* and *Candidatus_Solibacter* showed a significant negative correlation with AAN. *Bradyrhizobium* showed a highly significant positive correlation with TK. *Blastococcus* showed a highly significant negative correlation with AP and a significant negative correlation with soil TP, TK, EC, TON and NN. SU was highly significantly positively correlated with *Rhodanobacter* and *Gemmatimonas* and significantly negatively correlated with *Blastococcus* and *Candidatus_Solibacter*. *Bryobacter* was highly significantly positively correlated with SP activity and highly significantly negatively correlated with SNR.

For fungi (**Figures 7D, E**), *Mrakia* showed a highly significant positive correlation with soil pH and a significant positive correlation with soil SOM, AN and LON. *Heterocephalacria* showed a significant negative correlation with soil TP. *Fusarium* showed a highly significant negative correlation with NN and a significant negative correlation with AP and EC values. *Chaetomium* showed a significant negative correlation with soil TN. SU was significantly negatively correlated with *Fusarium*. *Mrakia* was significantly positively correlated with SP activity and negatively correlated with SNR activity.

From the above analyses, it can be concluded that *Gemmatimonas*, *Rhodanobacter* and *Mrakia* can effectively increase soil nitrogen content. These exhibit a reciprocal role with soil urease and protease activities, which in turn promote nitrification and ammonification, but are negatively correlated with the key denitrification enzyme nitrate reductase and reduce soil nitrogen loss. *Blastococcus* and *Fusarium*, on the other hand, had a limiting effect on soil nitrogen accumulation and soil nitrification and ammonification, and were positively correlated with nitrate reductase, the key denitrification enzyme, increasing soil nitrogen loss.

#### 3.5.5 Analysis of functional factors of soil microbial community based on the structural equation model

**Figure 8:**
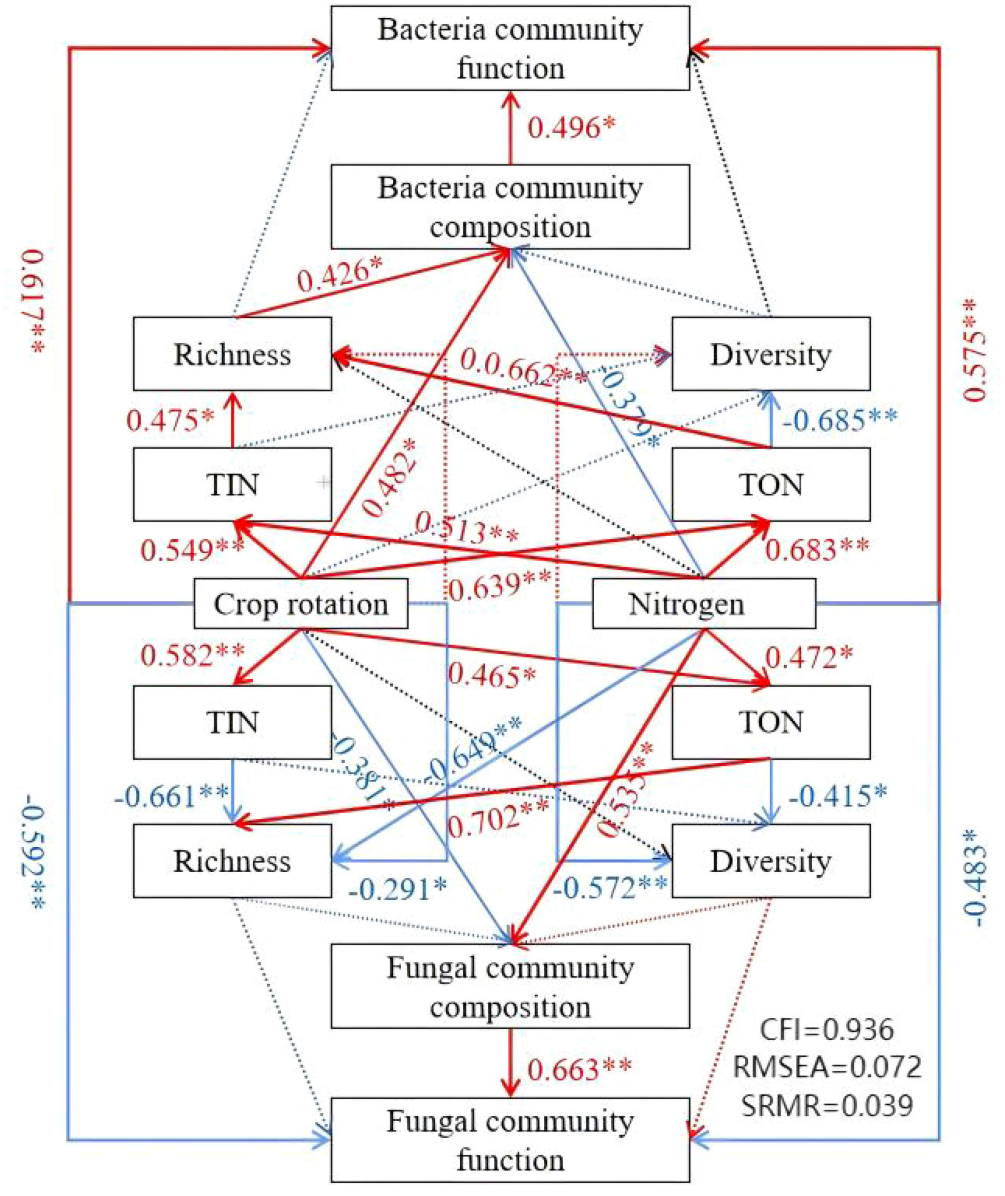
SEM-based functional analysis of the intricate interconnections among cultivation practices, nitrogen levels, and soil microbial communities. Red arrows indicate positive associations, while blue arrows depict negative relationships. Statistical significance is denoted by asterisks (*P < 0.05; **P < 0.01). TON, total organic nitrogen; TIN, total inorganic nitrogen.

The analysis results of the structural equation model (SEM) are presented in **Figure 8**. It indicated that both crop rotation and fertilization could significantly enhance soil TIN and TON, ranging from 32.11% to 57.02% (*P* < 0.05). The rhizospheric influence of nitrogen components was identified as the primary driving factor for soil microbial abundance. Crop rotation exerted a positive effect on the bacterial community composition and a negative effect on the fungal community composition. Interestingly, fertilizer addition showed the opposite pattern, with a negative effect on the former and a positive effect on the latter. Soil inorganic nitrogen content demonstrated a positive effect on bacterial community richness but a negative effect on fungal community richness, significantly impacting both fungal and bacterial community diversities. Soil organic nitrogen had a favorable influence on bacterial and fungal community richness, with a greater impact on bacteria than fungi. However, it had a deleterious impact on the diversity of both fungal and bacterial communities, with bacteria being affected more than fungi.

This study underscores that different forms of nitrogen exert significantly varying impacts on the compositions of bacterial and fungal communities (*P* < 0.05). As the proportion of soil TON components increased, the diversity of soil bacteria and fungi decreased, leading to alterations in their community compositions. Within this study, both bacterial and fungal community compositions exhibited positive effects on their respective community functionalities. The mechanisms through which crop rotation and fertilization influence microbial communities encompass an increase in inorganic and organic nitrogen content, thereby enhancing the abundance of soil microbial communities while reducing their diversity. This alteration in the community composition consequently enhances the functionalities of these microbial communities. Therefore, in subsequent research that employs models to predict soil nitrogen cycling, accounting for the connection between nitrogen forms and soil microbial community composition becomes imperative.

## 4 Discussion

### 4.1 Long-term crop rotation and nitrogen application-induced shifts in soil nitrogen forms

Within the context of soybean-maize rotation, maize often gains a competitive edge in soil nitrogen acquisition, as its nitrogen consumption exceeds that of soybeans (Gaudin et al., 2015). This, in turn, stimulates soybean’s nitrogen fixation capacity. Sainju (2022), in their study concerning the impact of rotation on soil physicochemical properties in black calcareous soil regions, corroborated a notable decrease of 15.5% in soil total nitrogen content following crop rotation. In the current work, the rotational practice significantly lowered the soil’s total nitrogen content during the maturation phase. This phenomenon primarily arose from the mutualistic interaction between maize and soybeans. During periods of low nitrogen availability, leguminous plants’ nitrogen fixation capacity adapts to enhance fixation rather than diminish it. Consequently, soybeans transfer nitrogen to maize, strengthening the nutrient translocation that augments maize’s nitrogen nourishment and stimulates its growth (Kätterer et al., 2019).

Within this study, the soil’s post-rotation levels of acid hydrolyzable nitrogen and nitrate nitrogen were noticeably greater than those of ammonium nitrogen. This divergence can be attributed to the transformation of ammonium nitrogen into acid hydrolyzable nitrogen and nitrate nitrogen during the nitrification process. A greater disparity between acid hydrolyzable nitrogen and nitrate nitrogen leads to higher availability of nitrate nitrogen for translocation (Ding et al., 2022). Hohman et al. (2021) reported enhanced nitrate nitrogen content in soybean-maize rotation systems (Hohman et al., 2021). Unlike continuous cultivation, the extended rotation of maize and soybean substantially elevates soil total organic nitrogen levels, with light fraction organic nitrogen content exerting the greatest influence. As compared to the MS0 and SS0 treatments, long-term fertilization had a considerable influence on total organic nitrogen content. Fertilization primarily elevated soil light fraction organic nitrogen, whose concentration increased with the duration of the rotation. The order of heavy fraction organic nitrogen content across treatments was: rotation > continuous cultivation, fertilized > non-fertilized, signifying that the combination of fertilization and crop rotation can enhance heavy fraction organic nitrogen. This outcome primarily stems from the differing cultivation practices. In conventionally tilled soils, nitrogen predominantly transforms into amide nitrogen, while amino sugar nitrogen and amino acid nitrogen prevail in rotated soils (Alizadeh et al., 2017).

### 4.2 The prolonged practice of crop rotation and nitrogen applications alters the structure of soil microbial communities

The dynamics of soil nitrogen reflect shifts in the overall microbial population within the soil. In the context of long-term rotation between rice and soybean, the bacterial composition at the genus level remains quite similar. The relative abundance of these has been consistently higher in rotational soil compared to continuous cropping, indicating that cultivation practices have a major influence on the distribution of bacterial genera. This could be attributed to the influence of alternating crops on bacterial composition (Fan et al., 2020). Microbial community analysis via PCA and correlation analysis revealed that after rotation, *Gemmatimonas*, *Rhodanobacter* and *Mrakia* significantly contributed to soil nitrogen content. This contribution was intertwined with soil urease and protease activities, consequently promoting nitrification and ammonification processes within the soil. However, there was a negative correlation with the key enzyme for denitrification, nitrate reductase, thereby reducing nitrogen loss. This is because *Gemmatimonas* and *Rhodanobacter* are aerobic bacteria, soil permeability is improved after crop rotation between legumes and grasses, and these two genera fix atmospheric nitrogen under low oxygen pressure (Zhang et al., 2019). The co-occurrence network model illustrated that crop rotation increased the number of soil bacterial edges, and the interrelationships between genera tended to be complex. The soil microbial community increased in species richness with increasing crop years, and the competitive relationship between species was weakened. This is because crop rotation improves the survival environment of the dominant genera in the soil, which in turn leads to the enhancement of their symbiotic relationship and the weakening of their competitive relationship (Soman et al., 2017).

Construction of a co-occurrence network model revealed that rotation increased the edges among soil bacteria, leading to a more complex interplay among phyla. As planting years grow, soil microbial communities display increased species richness and reduced inter-species competition. This shift is due to improved survival conditions for dominant bacterial phyla post-rotation, resulting in strengthened mutualistic relationships and reduced competitive interactions (Liu et al., 2023). Rotation effectively enhances the relative abundance of *Ascomycota* while decreasing that of *Basidiomycota*. *Basidiomycota*, being a large and complex fungal group, includes several plant pathogens like *Chaetomium* and *Fusarium*. These pathogens, which are major drivers of soybean root rot, often increase in abundance during continuous soybean cropping, potentially elevating the incidence of crop diseases (Zhou et al., 2018).

In this study, *Mrakia* in the *Ascomycota* effectively increased soil nitrogen content while simultaneously boosting the activities of soil urease and protease enzymes. This effect can be attributed to yeast symbionts in the soil, which proliferate around plant roots. Their gelatinous secretions enhance soil structure by increasing looseness, aeration, water retention, and nutrient preservation. This, in turn, decomposes nitrogen, phosphorus, potassium, and other immobilized elements in the soil, transforming them into nutrients that plants can directly absorb and utilize. As a result, the utilization efficiency of fertilizers is enhanced (Naumova et al., 2017).

### 4.3 Impact of long-term crop rotation on nitrogen form transformation: insights from microbial community dynamics and enzyme activities

In this study, the pivotal species and enzymatic activities associated with variations in nitrogen forms were identified using PCA analysis. Within the total nitrogen group, the proportions of heavy fraction organic nitrogen and alkali hydrolyzable nitrogen components were notably predominant in total organic nitrogen and inorganic nitrogen, respectively. This phenomenon is most likely due to the modulation of key microbial communities and enzymatic activities regulating the formation of heavy fraction organic nitrogen and alkali hydrolyzable nitrogen. alkali hydrolyzable nitrogen contains elements like ammonium nitrogen and nitrate nitrogen that are plausible nitrogen sources for plant uptake and constitute one of the most dynamic nitrogen reservoirs for crop growth (Brown et al., 2022). The fluctuation of alkali hydrolyzable nitrogen in the soil could be linked to variations in acid hydrolyzable nitrogen, given its significant presence within alkali hydrolyzable nitrogen and its role as a swiftly releasable fraction. *Proteobacteria* and *Acidobacteriota* as well as *Ascomycota* have previously been identified as members of the Dissimilatory Nitrate Reduction to Ammonium (DNRA) community (Saijai et al., 2016).

Crop rotation and nitrogen fertilization might induce ammonium reduction, curbing nitrogen loss via ammonia volatilization and thereby promoting ammonium nitrogen and nitrate nitrogen accumulation. The production of chitinase by Mrakia in the *Ascomycota* has been reported (Hu et al., 2022) and thus, chitinase decomposition of organic nitrogen products could add to the pool of ammonium nitrogen and nitrate nitrogen. Furthermore, recent reports indicate *Mrakia*’s participation in crop pathogen suppression, assistance in crop growth, and the accumulation of ammonium nitrogen and nitrate nitrogen components through enhanced root exudation (Challacombe et al., 2019). The significant influence of soil urease, protease, and nitrate reductase on the changes in ammonium nitrogen components was documented in the present work. Soil protease is engaged in the breakdown of chitin and lignin, key constituents of bacterial and fungal cell walls (Zhang et al., 2020). Following microbial cell wall shedding, decomposition generates low-molecular-weight organic compounds like free amino acids and amino sugars, contributing to the inorganic nitrogen pool. On the other hand, soil urease’s end product is NH_4_^+^, a precursor of ammonium nitrogen. However, when nitrogen saturation occurs, soil nitrate reductase may lead to a reduction in ammonium nitrogen and nitrate nitrogen content (Alizadeh et al., 2017).

In the MS1 treatment, ammonium nitrogen and nitrate nitrogen content significantly surpassed those of MS0 and SS0 treatments, suggesting that *Gemmatimonas, Rhodanobacter* and *Mrakia* excel in modulating alkali hydrolyzable nitrogen content compared to soil nitrate reductase. Certain fungal species have been reported to possess genes associated with amino acids (Frey et al., 2000), participating in nitrogen cycling. Their ability to produce an amino acid oxidase has also been observed (Isobe et al., 2014). The lower ammonia-producing capability following crop rotation is explained by decreased fungal richness relative to continuous cropping. Notably, in this study, Fungi demonstrate greater resilience to lower pH levels than bacteria in microbial communities (Zhang et al., 2021). Thus, the fungal-to-bacterial richness ratio rose with nitrogen fertilization due to the anticipated pH decrease caused by nitrogen input. This possibly elucidates the critical relevance of fungi in alkali hydrolyzable nitrogen morphological changes, as alkali hydrolyzable nitrogen is intricately tied to microbial metabolism (Ren et al., 2023).

## 5 Conclusion

This study’s findings reveal that under soybean-maize rotation conditions, both LON and ANN, as well as their constituent forms, namely AHN, AN, and NN, increased significantly. *Gemmatimonas, Rhodanobacter* and *Mrakia* could effectively increase soil nitrogen content, whereas *Blastococcus* and *Fusarium* increased soil nitrogen loss. It is critical to account for soil microenvironmental and functional factors in the investigation of soil nitrogen cycling across distinct rotation systems, given their direct impact on the alteration of soil nitrogen forms. The identification of nitrogen-promoting and nitrogen-depleting microbial taxa provides a basis for optimizing microbial inoculants or biocontrol strategies to enhance nitrogen use efficiency in rotation systems. While correlations between microbial taxa and nitrogen transformations were observed, experimental validation is needed to confirm causative mechanisms.

## Data Availability

The data presented in the study are deposited in the Zenodo repository. You can download, inspect, and further explore the relevant data via the following links: https://doi.org/10.5281/zenodo.17383398.
