# Supplementary material for: Long-term maize-soybean crop rotation: impacts on soybean yield, soil microbiota and nitrogen dynamics
Source: Front Plant Sci. 2025 Oct 27;16:1658885. doi: 10.3389/fpls.2025.1658885 (PMC12598005; doi:10.3389/fpls.2025.1658885)
Supplement: Supplementary file 1 [file Table1.docx]

**Table S1.** Basic physicochemical properties of soil under various treatments from 2020 to 2022. SOM: organic matter; TP: total phosphorus; TK: total potassium; TN: Total Nitrogen; SBD: Soil Bulk Density. MS1: Fertilized soybean-maize rotation, MS0: Non-fertilized soybean-maize rotation, SS1: Fertilized continuous soybean cultivation, and SS0: Non-fertilized continuous soybean cultivation.

| Years | Treatment | pH | EC  （uS cm^-1^） | SOM  （g kg^-1^） | TK  （g kg^-1^） | TP  （g kg^-1^） | TN  （g kg^-1^） | SBD  (g cm^-3^) |
| --- | --- | --- | --- | --- | --- | --- | --- | --- |
| 2020 | MS1 | 6.31±0.03 | 43.54±0.76 | 39.30±2.79 | 6.51±0.08 | 1.06±0.02 | 3.72±0.54 | 1.12±0.03 |
|  | MS0 | 6.11±0.10 | 18.20±0.88 | 49.99±3.47 | 6.41±0.17 | 1.13±0.03 | 3.31±0.48 | 1.09±0.05 |
|  | SS1 | 6.32±0.03 | 46.34±0.93 | 27.70±2.79 | 7.32±0.15 | 1.05±0.02 | 3.40±0.29 | 1.15±0.01 |
|  | SS0 | 6.28±0.07 | 22.30±1.71 | 30.36±5.52 | 7.54±0.10 | 1.27±0.03 | 3.29±0.88 | 1.14±0.05 |
| 2021 | MS1 | 6.25±0.05 | 40.25±1.25 | 37.26±3.92 | 6.62±0.17 | 1.14±0.02 | 3.77±0.48 | 1.12±0.08 |
|  | MS0 | 6.18±0.04 | 19.46±0.45 | 47.71±3.86 | 6.54±0.25 | 1.15±0.18 | 3.53±0.19 | 1.08±0.02 |
|  | SS1 | 6.28±0.24 | 44.43±2.17 | 28.35±2.01 | 7.38±0.21 | 1.07±0.05 | 3.39±0.02 | 1.17±0.09 |
|  | SS0 | 6.29±0.11 | 23.82±2.01 | 32.52±1.17 | 7.47±0.19 | 1.31±0.02 | 3.32±0.42 | 1.14±0.05 |
| 2022 | MS1 | 6.19±0.12 | 38.83±1.96 | 38.25±1.59 | 6.67±0.26 | 1.13±0.05 | 3.81±0.13 | 1.10±0.07 |
|  | MS0 | 6.21±0.05 | 21.07±1.01 | 46.19±2.24 | 6.58±0.19 | 1.19±0.08 | 3.50±0.24 | 1.08±0.01 |
|  | SS1 | 6.23±0.14 | 43.51±0.46 | 30.51±1.05 | 7.35±0.27 | 1.04±0.02 | 3.35±0.16 | 1.17±0.03 |
|  | SS0 | 6.34±0.02 | 25.95±2.42 | 31.44±2.59 | 7.82±0.37 | 1.15±0.09 | 3.84±0.21 | 1.15±0.06 |

**Table S2.** A PERMANOVA-test based on the PCA analysis. MS1: Fertilized soybean-maize rotation, MS0: Non-fertilized soybean-maize rotation, SS1: Fertilized continuous soybean cultivation, and SS0: Non-fertilized continuous soybean cultivation.

|  |  | Bacteria | | |  | Fungi | | |
| --- | --- | --- | --- | --- | --- | --- | --- | --- |
| Years | Pairs | R2 | p.value | p.adjusted |  | R2 | p.value | p.adjusted |
| 2020 | MS1vsMS0 | 0.217351 | 0.215 | 0.2152 |  | 0.223728 | 0.221 | 0.2212 |
|  | MS1vsSS1 | 0.474857 | 0.001 | 0.0061 |  | 0.372832 | 0.007 | 0.0427 |
|  | MS1vsSS0 | 0.459755 | 0.001 | 0.0054 |  | 0.438645 | 0.004 | 0.0216 |
|  | SS1vsSS0 | 0.359271 | 0.011 | 0.0165 |  | 0.368864 | 0.011 | 0.0165 |
|  | MS0vsSS1 | 0.392871 | 0.008 | 0.0121 |  | 0.390627 | 0.008 | 0.0121 |
|  | MS0vsSS0 | 0.418273 | 0.005 | 0.0091 |  | 0.357292 | 0.014 | 0.0255 |
| 2021 | MS1vsMS0 | 0.682972 | 0.001 | 0.0011 |  | 0.498203 | 0.001 | 0.0011 |
|  | MS1vsSS1 | 0.442837 | 0.002 | 0.0086 |  | 0.348273 | 0.002 | 0.0069 |
|  | MS1vsSS0 | 0.419273 | 0.007 | 0.0106 |  | 0.482776 | 0.001 | 0.0018 |
|  | SS1vsSS0 | 0.502832 | 0.001 | 0.0025 |  | 0.192738 | 0.238 | 0.5954 |
|  | MS0vsSS1 | 0.428271 | 0.006 | 0.0091 |  | 0.389271 | 0.015 | 0.0226 |
|  | MS0vsSS0 | 0.197283 | 0.199 | 0.1993 |  | 0.302819 | 0.035 | 0.0351 |
| 2022 | MS1vsMS0 | 0.699361 | 0.001 | 0.0011 |  | 0.415362 | 0.001 | 0.0011 |
|  | MS1vsSS1 | 0.659218 | 0.001 | 0.0011 |  | 0.358235 | 0.015 | 0.0165 |
|  | MS1vsSS0 | 0.552713 | 0.001 | 0.0027 |  | 0.333526 | 0.016 | 0.0432 |
|  | SS1vsSS0 | 0.142362 | 0.141 | 0.1409 |  | 0.082371 | 0.482 | 0.4817 |
|  | MS0vsSS1 | 0.409836 | 0.005 | 0.0089 |  | 0.362717 | 0.014 | 0.0249 |
|  | MS0vsSS0 | 0.369281 | 0.008 | 0.1594 |  | 0.322989 | 0.016 | 0.3188 |
